# Identifying Four Developmental Trajectories of Metabolic Syndrome and Their Influencing Factors: A Longitudinal Cohort Study of Railway Employees' Physical Examinations

**DOI:** 10.1155/ije/9237368

**Published:** 2025-11-14

**Authors:** Lin Jiang, Yuan Chen, Xiaona Cong, Hongwu Wang, Tao Jiang, Min Yang, Boao Xiao, Lishun Xiao, Yansu Chen

**Affiliations:** ^1^Shanghai Institute of Railway Disease Prevention and Control, China Railway Shanghai Bureau Group Co., Ltd., Shanghai 200071, China; ^2^School of Public Health, Xuzhou Medical University, Xuzhou 221004, Jiangsu, China; ^3^Key Laboratory of Human Genetics and Environmental Medicine, Xuzhou Medical University, Xuzhou 221004, Jiangsu, China; ^4^Department of General Surgery, The Affiliated Hospital of Xuzhou Medical University, Xuzhou 221006, Jiangsu, China

**Keywords:** developmental trajectories, influencing factors, latent growth mixture model, mixed-effect model, machine learning, metabolic syndrome

## Abstract

**Background:**

Metabolic syndrome (MetS) is one of the most common chronic disease complications and significantly increases the prevalence of chronic diseases. This study aims to identify different patterns of MetS development using longitudinal data and explore their influencing factors.

**Method:**

Based on the physical examination cohort of Shanghai railway workers, longitudinal data spanning 5 years (from January 1, 2019, to December 31, 2023) were collected to analyze the development trajectories of 1954 participants with MetS. Latent growth mixture model (LGMM) was employed to classify the development trajectories of MetS into distinct groups. Additionally, mixed-effect models were utilized to explore the influencing factors, and machine learning models were constructed for trajectory prediction.

**Results:**

The LGMM model classified patients into four groups: the progressively increasing group, the steadily increasing group, the progressively decreasing group, and the steadily decreasing group. Compared to the other three groups, the progressively increasing group exhibited the highest levels of weight, body mass index (BMI), heart rate, *γ*-glutamyltransferase, aspartate aminotransferase, alanine aminotransferase, uric acid, and white blood cell count. Conversely, compared to the other three groups, the group with progressive decreases showed the highest levels of systolic blood pressure, total bilirubin, direct bilirubin, urea nitrogen, fasting blood glucose, high-density lipoprotein cholesterol (HDL-C), and triglycerides (TGs). Mixed-effect models revealed that an increase in BMI and TG (OR > 1, *p* < 0.001) significantly increased the probability of being classified into the progressively increasing group, whereas HDL-C (OR < 1, *p* < 0.001) had the opposite effect. Variables selected through feature engineering were utilized to construct five machine learning prediction models, among which Random Forest (with an area under the curve, AUC = 0.852) demonstrated the best overall prediction performance and was therefore chosen to develop a MetS risk calculator based on Shiny.

**Conclusion:**

BMI, TG, and HDL-C were the key to influence the developmental trajectories of MetS. Therefore, these three indicators should be closely monitored, and the progression of MetS can be controlled by adjusting dietary patterns.

## 1. Introduction

According to the definition of the World Health Organization (WHO), comorbidity refers to the coexistence of two or more chronic conditions in an individual [1]. Compared to a single chronic condition, chronic comorbidities not only significantly increase the risks of physical functional limitations, disability, and mortality for patients, reducing their labor productivity and quality of life, but also markedly elevate the complexity and difficulty of prevention and treatment, thereby increasing healthcare expenditures for families and society. The incidence of chronic comorbidity is influenced not only by population aging and lifestyle but also closely associated with socioeconomic conditions and disparities in healthcare systems [2]. With population aging and changes in lifestyle, the incidence of chronic comorbidity overall demonstrates an upward trend [3].

Metabolic syndrome (MetS) is one of the most common chronic complications, referring to a state in which multiple metabolic factors such as obesity, hypertension, dyslipidemia, and abnormal glucose metabolism cluster in the human body [4]. MetS serves as a contributing factor for chronic diseases such as cardiovascular and cerebrovascular diseases and diabetes. If not prevented and treated promptly, it can progress to more severe cardiovascular and cerebrovascular events or even death [4]. With the development of socioeconomic conditions and the improvement of people's material living standards, the incidence of MetS has been increasing year by year. Currently, the prevalence rate among adults aged 20 and above in China is 31.1%, which means that approximately 450 million people in China are affected by MetS [5]. MetS has become a major public health issue that seriously jeopardizes human health.

The special group of railway workers is characterized by shift work involving night shifts and prolonged periods of sitting or standing. The prevalence of MetS among this group is significantly higher than that in the general population due to the notable increase in risk associated with night shift work. In terms of gender differences, male workers generally have a higher prevalence than female workers. Furthermore, railway workers exhibit a “three lows and one high” characteristic in their awareness of MetS, namely, low awareness, low treatment rates, low rates of achieving treatment goals, and high prevalence [6]. Railway workers with MetS may face an increased risk of accidents at work, which can lead to issues such as impaired vision, numbness in the hands and feet, dizziness, and palpitations. These problems may further compromise operational safety among workers. Therefore, popularizing knowledge about MetS and providing timely prevention and treatment not only help the railway system reduce medical and labor costs but also enable the provision of targeted medical services to individual railway workers, thereby facilitating a more rational allocation and utilization of health resources within the railway system.

Many scholars have conducted extensive researches on the etiology [7], pathophysiological mechanisms [8], diagnostic criteria, and treatment strategies [9] of MetS, and different individuals may have different patterns of MetS development. Longitudinal measurements help to identify patterns, trends, or cyclical changes in disease, thereby revealing underlying group characteristics or classifications, providing important support for personalized early warning, treatment, clinical management, and decision-making [10]. The latent growth mixture model (LGMM) is a statistical model used to study intraindividual developmental trajectories, which allows groups to be classified into categories based on heterogeneity, thus identifying heterogeneous developmental trajectories between groups and estimating the characteristics and differences at each stage [11–13].

This study, based on 5 years of longitudinal physical examination data from railway workers, utilized the LGMM to identify the development trajectories of MetS and determine the characteristic parameters of the trajectory groups. Furthermore, mixed-effect models were employed to delve deeper into the key factors influencing the development of these trajectories. Additionally, we applied machine learning to predict metabolic progression and developed a website for metabolic development prediction. This research provides theoretical support and practical guidance for the prevention and control of MetS among railway workers.

## 2. Materials and Methods

### 2.1. Ethical Approval and Informed Consent

This study was conducted in accordance with the Declaration of Helsinki. It was approved by the Ethics Committee of Xuzhou Medical University (Grant No. XZHMU-2023626). All participants provided the informed consent. The informed consents were obtained from each individual by a mobile electronic questionnaire containing the study information. The authors had no access to information that could identify individual participants during or after data collection.

### 2.2. Data Collection and Selection of Participants

The study was conducted within the framework of a physical examination cohort of railway workers over a 5-year period, from January 1, 2019, to December 31, 2023, which was collected by the Shanghai Railway Disease Prevention and Control Institute of China Railway Shanghai Group Co., Ltd. The dataset included age, gender, height, weight, body mass index (BMI), heart rate, systolic blood pressure (SBP), diastolic blood pressure (DBP), fasting blood glucose (FBG), triglycerides (TGs), total cholesterol, high-density lipoprotein cholesterol (HDL-C), low-density lipoprotein cholesterol (LDL-C), hemoglobin, white blood cell count (WBC), red blood cell count (RBC), alanine aminotransferase (ALT), aspartate aminotransferase (AST), *γ*-glutamyltransferase (GGT), direct bilirubin, total bilirubin, albumin, uric acid, blood urea nitrogen (BUN), and urine pH. BMI was calculated by weight/height [2] (kg/m^2^).

**Figure 1:**
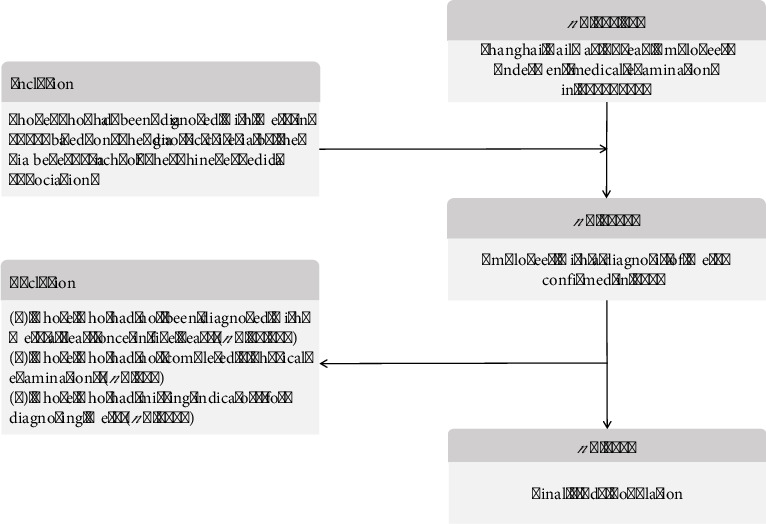
Flowchart for the selection of participants.

A total of 112,157 employees made annual medical examination and those diagnosed with MetS in 2019 were included in this study. The diagnostic criteria for MetS adopted from the version developed by the Diabetes Branch of the Chinese Medical Association, which included ① overweight or obesity (BMI ≥ 25.0), ② hyperglycemia (FBG ≥ 6.1 mmol/L and/or those who had been diagnosed with diabetes mellitus), ③ hypertension (SBP/DBP ≥ 140/90 mm/Hg, and/or those who had been diagnosed with hypertension), ④ dyslipidemia (fasting blood TG ≥ 1.7 mmol/L, and/or fasting HDL-C < 0.9 mmol/L in male population and < 1.0 mmol/L in female population), and MetS was diagnosed when three or all of the above four components were present. The exclusion criteria for this study were (1) those who had not been diagnosed with MetS at least once in 5 years, (2) those who had not completed five physical examinations, and (3) those who had missing evaluation indicators for diagnosing MetS. Eventually, 1954 individuals were included in this study, and the inclusion and exclusion processes are shown in Figure 1.

### 2.3. Data Processing

For repeated measurements of the same individual who had two physical examinations in the same year, the average of the two physical examination measurements was taken; missing values for continuous variables were filled in by median interpolation, and missing values for categorical variables were filled in by last observation carried forward; the boxplots were used to identify outliers, and any value less than the 1st percentile or greater than the 99th percentile was replaced with the 1st percentile or 99th percentile, respectively.

### 2.4. Continuous Metabolic Syndrome Severity Score (cMetS-S)

Confirmatory factor analysis (CFA) was used to fit BMI, SBP, HDL-C, TG, and FBG, and cMetS-S was calculated separately according to gender, which was mentioned in many previous studies [14–16]. To perform CFA analysis, since systolic and diastolic blood pressures are highly correlated [17], we chose to only include systolic blood pressure in CFA as it is more strongly associated with insulin resistance [18]. Based on studies demonstrating the validity of MetS severity scores based on both BMI and waist circumference in predicting future coronary heart disease and Type 2 diabetes mellitus (T2DM), and that both perform similarly in most cases, and that BMI-based scores may be advantageous in predicting T2DM, we chose BMI for the CFA [15].

### 2.5. LGMM

LGMM is a technique that classifies patients by calculating the posterior probability of belonging to a category and assigning them to the category with the highest probability [19]. This study used the *R* package “lcmm” to identify the trajectory of cMetS-S over time using a random intercept model with the time of study year as the variable. To assess the potential nonlinear characteristics of cMetS-S over time, we used a squared natural linear equation for the study year with a 5-year time points in the fixed-effects part. Due to the limited sample size of the study, the number of trajectories was restricted to a maximum of five groups, and Akaike Information Criterion (AIC), Bayesian Information Criterion (BIC), log-likelihood, and average posterior probability (APP) were used to assess the effectiveness of model fitting. The smaller the AIC and BIC values, the better the model fitted; the log-likelihood measured the probability that the model generated the observed data, and the larger the value, the better the model fitted; APP was the probability that the samples were classified into each trajectory group, and the average probability of each group was greater than 0.7, which indicated that the classification effect was better [19–22].

### 2.6. Mixed-Effect Model

This study employed the mixed-effect model to compare participants of different trajectory groups, aiming to explore the associations between trajectories and independent variables. Furthermore, we calculated the coefficients and odds ratios (ORs) of the fixed effects of these associations, which allowed us to assess the relationship between the variables and the trajectories. The mixed-effect models were implemented using the *R* package “lme4.”

### 2.7. Machine Learning

Using the final trajectory groups as the outcome classification labels, feature selection was conducted by the Boruta algorithm. Subsequently, five machine learning models were developed: extreme gradient boosting (XGBoost), logistic regression, *k*-nearest neighbors (KNN), random forest, and support vector machine (SVM). The dataset was randomly divided into a training set (70%) and a test set (30%) to construct prediction models for MetS trajectory progression. Model performance was evaluated on the test set, with the area under the curve (AUC) of receiver operating characteristic (ROC) serving as the primary metric of discriminative ability. Higher AUC values indicate superior classification performance. To facilitate clinical translation, we developed an interactive risk calculator via the shiny package. Users can input their clinical parameters through a web interface to obtain (1) the predicted probability of belonging to the high-risk trajectory group and (2) a variable importance plot generated by the optimal model.

All analyses were conducted using *R* software (version 4.3.0) with the following packages: “xgboost” (XGBoost), “stats” (logistic regression), “class” (KNN), “randomForest” (random forest), “e1071” (SVM), and “shiny” (web application development). Hyperparameter tuning and model fitting were performed through a 10-fold cross-validation using the “caret” package.

### 2.8. Statistical Analysis

The study conducted analyses using SPSS.26 and R-4.3.1 software. Line graphs were plotted through OrignPro 2022 to describe the trend of characteristics over time for different trajectory groups. Mean ± standard deviation (SD) was used to describe continuous variables, and constitutive ratios were used to describe categorical variables, and statistical differences in variables among trajectory groups were implemented by analysis of variance (ANOVA) or χ^2^ test, and differences between two groups were conducted by the least significant difference method. A two-sided *p* value ≤ 0.05 was considered statistically significant.

## 3. Results

### 3.1. Description of Participants' Characteristics

This study described the characteristics of 1954 participants with MetS at baseline in 2019 and found that their mean age was 47.46 ± 7.35 years, 98% of them were male, 97% of them had overweight or obesity, 51% of them had hyperglycemia, 86% of them had hypertension, and 94% of them had dyslipidemia (Table 1).

The specific physical examination characteristics of the study population at baseline will be reflected in the supporting information. Among the general physical indicators, blood pressure and heart rate, blood glucose and blood lipids, blood routine, liver function, renal function, and urine indicators, the mean values exceeding the normal range include weight, BMI, SBP, DBP, FBG, TG, total cholesterol, GGT, and uric acid (Table S1).

### 3.2. Classification of MetS Developmental Trajectories

**Figure 2:**
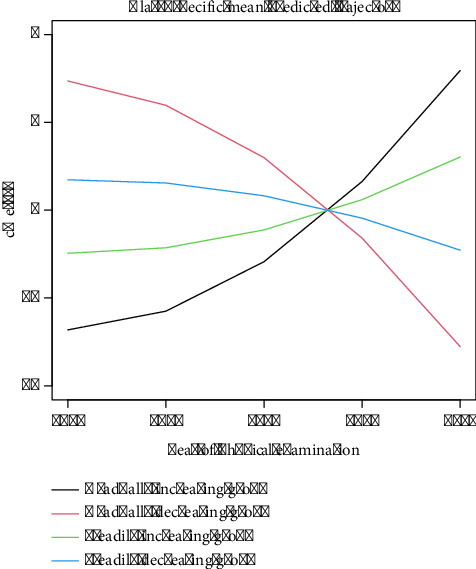
Classification chart of MetS developmental trajectories.

In LGMM, the number of trajectory groups was searched between 1 and 5, and the optimal number was determined by AIC, BIC, log-likelihood, and APP. When the trajectories were classified into four groups, the model exhibited the lowest AIC and BIC values, a relatively high log-likelihood value, and APP being greater than 0.7, indicating that the four-group classification provided the optimal fit and classification performance for the data (Table S2). Therefore, we categorized the MetS trajectories into four distinct groups based on their characteristics: gradually increasing group (*n* = 139, 7.1%), steadily increasing group (*n* = 791, 40.48%), gradually decreasing group (*n* = 204, 10.4%), and steadily decreasing group (*n* = 820, 41.96%). Given that a higher MetS value signifies more severe disease, Figure 2 illustrates that the decreasing trajectory groups exhibited a trend of gradual MetS improvement over time, whereas the increasing trajectory groups demonstrated a trend of progressive MetS deterioration.

### 3.3. Comparison of Between Groups of MetS Developmental Trajectories

Using ANOVA (for continuous variables) or *χ*^2^ tests (for categorical variables) revealed significant between-group differences (*p* < 0.05) in gender distribution and the following metabolic parameters: gender, age, weight, BMI, SBP, DBP, heart rate, FBG, TG, HDL-C, LDL-C, hemoglobin, WBC, ALT, AST, GGT, total bilirubin, direct bilirubin, total protein, urea nitrogen, and uric acid. To further elucidate the differences between groups, we employed the least significant difference method for pairwise comparisons.

At the baseline, the gradually increasing group admitted the highest age and heart rate, followed by the steadily increasing, gradually decreasing, and steadily decreasing groups. And the gradually increasing group owned the highest weight, BMI, TG, hemoglobin, RBC, WBC, ALT, AST, GGT, total protein, and creatinine, followed by the steadily increasing, steadily decreasing, and gradually decreasing groups. Conversely, the gradually decreasing group had the highest SBP, DBP, HDL-C, LDL-C, total bilirubin, and direct bilirubin, followed by the steadily decreasing, steadily increasing, and gradually increasing groups. Concerning total cholesterol, the gradually decreasing group had the highest value, followed by the gradually increasing, steadily decreasing, and steadily increasing groups. Besides, the gradually increasing group had the highest creatinine and the lowest BUN among the four groups. In summary, these findings revealed that the gradually increasing group always had highest levels of physiological and biochemical indicators that promote the development of MetS, including age, weight, BMI, heart rate, TG, in addition to the highest levels of platelet erythrocyte counts, white blood cell counts, albumin, and hepatic indices AST, ALT, and GGT. Blood pressure, FBG, total cholesterol, HDL-C, LDL-C, direct bilirubin, total bilirubin, and BUN were at the highest levels in the gradually decreasing group (Table 2).

**Figure 3:**
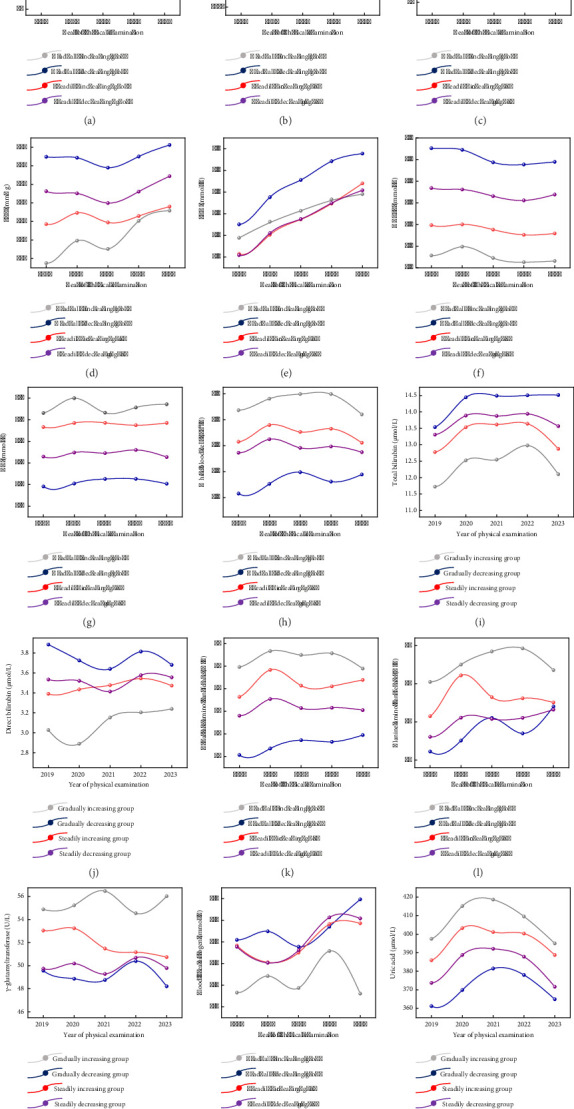
Line graphs of variables over time for the MetS developmental trajectory groups. (a) Body weight; (b) BMI; (c) heart rate; (d) SBP; (e) FBG; (f) HDL-C; (g) TG; (h) WBC; (i) total bilirubin; (j) direct bilirubin; (k) AST; (l) ALT; (m) GGT; (n) BUN; (o) uric acid.

Line graphs were used to visualize the temporal changes in physical examination variables across different trajectory groups (see Figure 3). The results revealed distinct patterns in key indicators among these four groups. For the gradually increasing group, weight, heart rate, BMI, WBC, hepatic enzymes (AST, ALT, GGT), and uric acid were significantly higher compared to other groups, while SBP, total bilirubin, direct bilirubin, and urea nitrogen were significantly lower compared to other groups. FBG and TG exhibited significant increasing trends over time (*p* < 0.01). Other variables, including weight, BMI, SBP, HDL-C, WBC, total bilirubin, direct bilirubin, AST, ALT, GGT, and blood urea nitrogen, remained stable (*p* > 0.05). Concerning the gradually decreasing group, SBP, FBG, HDL-C, TG, total bilirubin, and direct bilirubin were significantly higher compared to other groups, while weight, BMI, WBC, hepatic enzymes (AST, ALT, GGT), and uric acid were significantly lower compared to other groups. Similar to the gradually increasing group, FBG and TG showed significant increases over time (*p* < 0.01), whereas other variables remained stable (*p* > 0.05). Notably, the gradually increasing group and the gradually decreasing group exhibited diametrically opposed trends in most physical examination indicators. For example, TG levels increased by 12.7% annually in the gradually increasing group (Figure 3(c)), while AST levels exceeded the upper limit of normal (40 U/L) in 68% of this group (Figure 3(d)). These findings suggested that the trajectory groups have distinct patterns of metabolic and inflammatory changes, highlighting the importance of personalized monitoring and intervention strategies.

### 3.4. Influencing Factors for Developmental Trajectories of MetS

With the gradually decreasing group as the reference, mixed-effect modeling analysis revealed significant associations between several variables and the trajectories of MetS over time (*p* < 0.05). Specifically, an increase in BMI or TG (ORs > 1, *p* < 0.05) was associated with elevated odds of being attributed to an increasing trajectory. Conversely, an increase in FBG or HDL-C (ORs < 1, *p* < 0.05) was associated with reduced odds of being attributed to an increasing trajectory. Additionally, an increase in AST (ORs > 1, *p* < 0.01) was associated with elevated odds of being attributed to an increasing trajectory. An increase in urine pH (OR = 1.19, *p* < 0.01) was associated with elevated odds of falling into the steadily decreasing group (Table 3).

Using either the steadily decreasing group or the steadily increasing group as the reference, mixed-effect modeling analysis revealed that the associations between BMI, FBG, TG, HDL-C, urea nitrogen, total serum cholesterol, and the trajectories of MetS were statistically significant over time (*p* < 0.05). Specifically, increases in BMI, FBG, and TG (ORs > 1, *p* < 0.05) were associated with elevated odds of being attributed to the gradually increasing group, whereas increases in HDL-C, urea nitrogen, or total serum cholesterol (ORs < 1, *p* < 0.01) were associated with reduced odds of being attributed to this group (Table 4). In conclusion, increases in BMI and TG were associated with the malignant progression of MetS, while an increase in HDL-C was strongly associated with the remission of MetS.

### 3.5. Predictive Model for Developmental Trajectories of MetS

**Figure 4:**
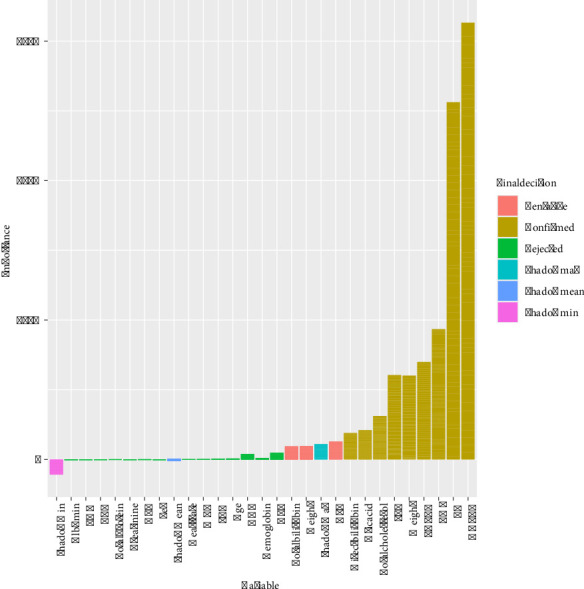
The results of feature variable selection using the Boruta algorithm.

To enhance predictive performance, we combined the gradually decreasing and steadily decreasing groups as a MetS improvement group, as well as the gradually increasing and steadily increasing groups as a MetS deterioration group, to serve as the prediction outcomes. This approach leverages the observed patterns in these trajectories to more accurately predict the development of MetS. Firstly, the Boruta algorithm was adopted to selecting features with significant importance for prediction, and it was implemented with a stringent significance threshold (*p* < 0.01) to ensure the robustness. In the Boruta algorithm, shadowMax, shadowMean, and shadowMin are key statistics used to evaluate feature importance. shadowMax represents the maximum importance score obtained by shadow features across multiple iterations, serving as a benchmark to determine whether a real feature is significantly more important than random features. shadowMean is the average importance score of the shadow features, reflecting the typical importance level of these random features. shadowMin is the minimum importance score of the shadow features used to assess the lowest level of importance among them. These statistics are calculated using shadow features generated by randomly shuffling the values of the original features, helping the algorithm distinguish whether the importance of real features is statistically significant. If the *Z* value of a true feature is significantly higher than the maximum *Z* value of the shadow feature in multiple independent tests, the true feature is marked as “important” (confirmed), also known as an acceptable variable. Otherwise, it is marked as “unimportant” (rejected), also known as unacceptable variables. Acceptable variables are variables that are retained during the feature selection process and are considered to contribute to the performance of the model. Unacceptable variables are excluded from the final feature selection by the algorithm because they fail to show predictive power for the target variable during the feature selection process. To improve the robustness of the feature selection and prevent overfitting, we incorporated a cross-validation approach into the Boruta feature selection process. Specifically, the dataset was split into training and testing subsets (70% for training and 30% for testing). Boruta's feature selection process was conducted solely on the training set, and the identified significant features were used to train models. The results revealed that weight, BMI, SBP, TG, total cholesterol, HDL-C, LDL-C, uric acid, and direct bilirubin were identified as important features (Figure 4). Notably, HDL-C and TG emerged as the most influential predictors of MetS progression, highlighting their critical role in the model.

**Figure 5:**
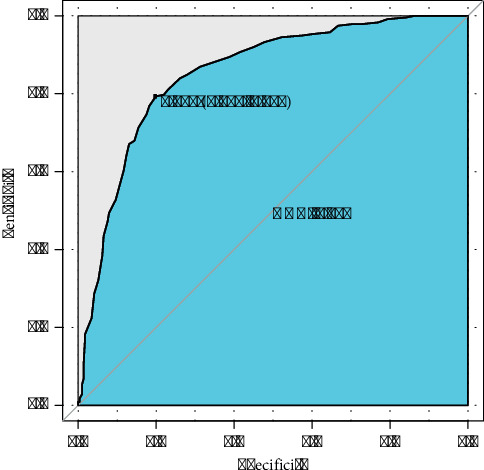
The ROC curve for the random forest model in the test set.

Nine feature variables identified by the Boruta algorithm were incorporated into the machine learning models. The performance of these models was evaluated using sensitivity, specificity, Kappa consistency, F1-score, accuracy, precision, recall, and AUC values. In the test set, the random forest model demonstrated the highest accuracy (0.812), Kappa (0.623), specificity (0.847), precision (0.848), and F1-score (0.813) (Table 5). These results indicate that the random forest model outperformed other models across the evaluation metrics. Notably, the difference in evaluation metrics between the training and test sets of the random forest model was very small, confirming that there existed no overfitting or underfitting. Therefore, the random forest model was selected as the predictive model for MetS improvement and deterioration groups. The ROC curve for the test set is provided in Figure 5, illustrating the model's robust ability to distinguish between the two groups. The AUC value (0.852) highlighted the model's capacity to accurately classify individuals into MetS improvement and deterioration groups, thereby supporting early intervention and management strategies.

Finally, we developed a random forest model-based risk predictor using the “shiny” package, an interactive web application designed to facilitate real-time predictions of MetS progression. This tool is particularly relevant for occupational health assessments, such as the annual physical examinations of railway workers, where early detection and intervention can significantly improve health outcomes.

Additionally, lifestyle-related determinants were captured in the same cohort of railway employees with MetS. After rigorous quality control, 1073 valid questionnaires were retained for analysis (Table S3). Variables included the length of service, smoking status, metabolic equivalents of task (METs), alcohol consumption, marital status, living arrangement (living alone vs cohabiting), predominant work environment (indoor vs outdoor), shift pattern (day-only vs rotating night shift), daily tea intake, daily water consumption, spicy-food preference, estimated daily salt and oil intake, late-night eating habits, and familial hypertension history.

Following inclusion of all 1073 records, Boruta feature selection repeatedly relegated every lifestyle variable to the “unimportant” stratum (Figure S1). To formally quantify the predictive utility of these factors for MetS trajectory, we nevertheless retained them in the machine learning pipeline. SHAP summary plots (Figure S2) and bee-swarm plots (Figure S3) were generated, and model-performance metrics are summarized in Table S4. Across algorithms, lifestyle covariates contributed negligibly to predictive accuracy, most likely because of a high proportion of missing responses that undermined representativeness.

To definitively appraise the prognostic value of lifestyle behavior on metabolic trajectories, questionnaire accrual will continue until an adequate, fully populated sample is achieved; the final model will be predicated on these augmented data.

During the railway workers' physical examinations, medical staff can log into this web application and enter actual values for key health indicators, including weight, BMI, systolic blood pressure, triglycerides, total cholesterol, HDL-C, LDL-C, direct bilirubin, and uric acid. Then, the application can instantly display the probability of the individual's condition worsening, along with the ROC curve and the feature importance chart. These visualizations provide a comprehensive overview of the individual's risk profile and help identify the most influential factors contributing to MetS. The risk calculator is available at https://plotting.shinyapps.io/prediction/.

## 4. Discussion

MetS has long been recognized as a cluster of metabolic abnormalities associated with an increased risk of cardiovascular disease, T2DM, and other chronic conditions. However, the presence of MetS alone does not fully predict the global cardiovascular disease risk [23]. Recent studies have shown that the trajectories of MetS are related to the prognosis in oncology patients and the incidence of multiple tumors or diabetes in the general population [16, 24]. Additionally, research has highlighted the association between BMI trajectories and the incidence of MetS or diabetes mellitus in both general and adolescent populations [25]. Despite these findings, there remains a gap in understanding the developmental trajectories and influencing factors of MetS specifically within patient populations [26–28].

The present study explored four development trajectories of MetS including gradually increasing, steadily decreasing, steadily increasing, and steadily decreasing based on an employee physical examination cohort and found that there was a significant difference in the severity of MetS among these trajectory patterns. Specifically, the patients of the gradually increasing group were the oldest and heaviest and had the highest levels of BMI, TG, leukocytes, ALT, AST, GGT, and uric acid, while the ones of the gradually decreasing group had the highest levels of SBP, FBG, HDL-C, total bilirubin, and direct bilirubin.

The identification of these diverse trends through line charts suggests the presence of multifaceted functional abnormalities among railway workers. This finding underscores the need for a comprehensive assessment and management approach. The trajectory classification not only replaces the traditional yes–no dichotomy of disease but also allows for dynamic consideration of changes in disease progression. For instance, individuals with MetS component levels close to the corresponding thresholds (within the normal range) but without associated symptoms may not be clinically diagnosed or treated for MetS. However, they may actually be at a higher risk for cardiovascular disease. In such cases, these individuals can be identified by their higher MetS scores and receive further clinical investigations or lifestyle interventions to reduce MetS scores and cardiovascular disease risk. This approach results in a more accurate diagnosis of an individual's metabolic health, facilitating long-term patient monitoring and better predicting the course of the disease.

In this study, we used mixed-effect model to explore the influence factors over time on the developmental trajectories of MetS and found that BMI, FBG, TG, and HDL-C were particularly important. Previous study has showed a strong association between a sustained high BMI trajectory in childhood and a significantly increased risk of developing MetS in midlife [27]. TG is closely related to MetS and is the main form of lipid storage in adipose tissue, where carbohydrate intake in excess of one's needs is stored as triglycerides in the body's adipose tissue [29]. HDL-C is associated with insulin resistance and that improving insulin resistance can increase the levels of HDL-C^30^. AST has been recognized as a marker of liver injury, including a wide range of etiologies from viral hepatitis to fatty liver [30]. Increasing incidence of MetS and cardiovascular disease worldwide suggests that AST is a strong predictor of T2DM, coronary artery disease, risk of atherosclerotic thrombosis, and overall risk of metabolic disease [30]. Urine pH was found to decrease progressively with increasing features of MetS, and testing fasting urine pH may be a practical screening tool for MetS [31]. MetS can lead to extra burden on the kidneys, resulting in impaired renal function and impaired excretion of nitrogenous wastes, such as urea nitrogen, from the body, leading to increased urea nitrogen levels in the blood [32, 33]. In conclusion, our study found that an increase in BMI and TG would significantly increase the risk of MetS severity, and an increase in HDL-C would significantly decrease the risk of MetS severity. Therefore, these three indicators should be strictly monitored and the development of MetS should be controlled by effective means such as weight control and adjustment of dietary patterns.

Through the analysis of the incidence and developmental trajectories of MetS among railway workers, as well as their influencing factors, we constructed predictive models to predict whether an individual's MetS will belong to the improvement group or deterioration group at baseline. Our findings provide a scientific basis for the monitoring, early warning, prevention, and intervention of MetS, thereby safeguarding workers' health, preventing adverse outcomes in affected individuals, and improving their quality of life. The overarching goal of our research is to enable individuals to foresee their future metabolic trends by accessing their physical examination data. Utilizing machine learning models, we predict the disease progression patterns of railway workers based on their initial physical examination characteristics. These predictions can guide workers in developing specific plans for managing MetS and serve as a wake-up call for those at risk.

This study also has several limitations. First, although this study utilizes longitudinal physical examination data from railway workers and employs advanced statistical models to explore the developmental trajectories and potential influencing factors of MetS, the findings may not be representative when extrapolated to the general population. This limitation arises due to the occupational specificity of the data and the observed gender imbalance, which may affect the generalizability of our results. Second, there may also be some unmeasured confounding that impact the progression of MetS; thus, there is likely residual bias.

In conclusion, using a longitudinal physical examination cohort of railway employees, this study explored the developmental trajectories of MetS and identified key factors influencing these trajectories over time. These findings underscore the importance of regular monitoring and early intervention in managing MetS within this occupational group. Future research should aim to validate these findings in broader populations and explore additional biomarkers or lifestyle factors that may further enhance our understanding and management of MetS.

## Figures and Tables

**Table 1:** The characteristics of the 1954 participants with MetS.

Characteristics	Values (*n* = 1954)
Age (years), mean ± SD	47.46 ± 7.35
Gender, *n* (%)	
Male	1911 (98)
Female	43 (2)
Overweight or obesity, *n* (%)	
Yes	1903 (97)
No	51 (3)
Hyperglycemia, *n* (%)	
Yes	983 (50)
No	971 (49)
Hypertension, *n* (%)	
Yes	1675 (86)
No	279 (14)
Dyslipidemia, *n* (%)	
Yes	1828 (94)
No	126 (6)

**Table 2:** Comparison of MetS development trajectory groups at baseline.

Characteristics	Gradually decreasing group (*n* = 204)	Steadily decreasing group (*n* = 820)	Steadily increasing group (*n* = 791)	Gradually increasing group (*n* = 139)	*p* values
Sex					0.656
Male	197	803	775	136	
Female	7	17	16	3	
Age (years)	45.63 ± 8.10	43.91 ± 8.15^a^	48.02 ± 7.06^ab^	50.02 ± 6.37^abc^	< 0.001
Weight (kg)	82.00 ± 8.20	83.34 ± 8.70^a^	86.07 ± 9.40^ab^	88.60 ± 9.30^abc^	< 0.001
Height (m)	1.72 ± 0.057	1.71 ± 0.057	1.72 ± 0.057	1.72 ± 0.056	0.695
BMI (kg/m^2^)	27.58 ± 1.75	28.03 ± 2.10^a^	28.77 ± 2.34^ab^	29.53 ± 2.36^abc^	< 0.001
SBP (mmHg)	152.90 ± 14.09	149.20 ± 13.80^a^	145.7 ± 13.79^ab^	141.50 ± 13.49^abc^	< 0.001
DBP (mmHg)	95.91 ± 9.77	95.84 ± 9.50	94.07 ± 10.00^ab^	92.50 ± 8.86^ab^	< 0.001
Heart rate (/min)	78.23 ± 10.01	77.91 ± 9.57^a^	78.23 ± 9.23	78.71 ± 10.02^ac^	0.049
FBG (mmol/L)	6.50 ± 1.05	6.22 ± 1.13^a^	6.23 ± 1.14^a^	6.38 ± 1.19^abc^	0.006
TG (mmol/L)	1.96 ± 0.906	2.69 ± 0.974^a^	3.33 ± 1.195^ab^	3.65 ± 1.238^abc^	< 0.001
Total cholesterol (μmol/L)	5.35 ± 0.88	5.25 ± 0.90^a^	5.10 ± 0.89^ab^	5.31 ± 0.94^abc^	< 0.001
HDL-C (mmol/L)	1.45 ± 0.22	1.27 ± 0.20^a^	1.10 ± 0.19^ab^	0.96 ± 0.19^bc^	< 0.001
LDL-C (mmol/L)	2.99 ± 0.75	2.98 ± 0.76^a^	2.75 ± 0.77^ab^	2.48 ± 0.79^abc^	< 0.001
Hemoglobin (g/L)	156.42 ± 9.79	157.56 ± 9.59^a^	158.26 ± 10.22^a^	159.59 ± 9.88^abc^	0.003
RBC (10^12^/L)	5.03 ± 0.42	5.11 ± 0.43^a^	5.15 ± 0.46^ab^	5.18 ± 0.45^abc^	< 0.001
WBC (10^9^/L)	7.36 ± 1.35	7.42 ± 1.30^a^	7.41 ± 1.36^a^	7.49 ± 1.33^abc^	< 0.001
ALT (U/L)	22.23 ± 6.15	22.60 ± 6.55	23.14 ± 6.67^a^	24.04 ± 6.65^abc^	< 0.001
AST (U/L)	26.12 ± 11.58	29.60 ± 13.03^a^	31.27 ± 13.66^ab^	33.92 ± 12.51^abc^	0.025
GGT (U/L)	49.56 ± 26.68	49.74 ± 25.26	53.04 ± 25.64^ab^	54.88 ± 28.03^abc^	0.016
Total bilirubin (μmol/L)	13.53 ± 4.68	13.30 ± 4.38^a^	12.77 ± 4.33^ab^	11.72 ± 3.85^abc^	< 0.001
Direct bilirubin (μmol/L)	3.88 ± 1.34	3.53 ± 1.30^a^	3.39 ± 1.36^a^	3.02 ± 1.32^abc^	< 0.001
Total protein (g/L)	73.62 ± 4.35	74.24 ± 4.31	74.11 ± 4.27^ab^	74.95 ± 4.35^abc^	0.040
Albumin (g/L)	45.52 ± 3.01	45.99 ± 2.87	45.91 ± 2.76	46.24 ± 2.69	0.088
BUN (mmol/L)	5.20 ± 1.02	5.17 ± 1.14	5.18 ± 1.14	4.96 ± 1.17^abc^	0.048
Uric acid (μmol/L)	361.16 ± 62.56	373.70 ± 64.37^a^	385.80 ± 61.13^ab^	379.48 ± 63.13^abc^	< 0.001
Creatinine (μmol/L)	69.51 ± 12.09	71.04 ± 12.26^a^	71.34 ± 12.15^a^	72.04 ± 13.04^a^	0.045
Urine pH	5.61 ± 0.64	5.63 ± 0.63	5.64 ± 0.59	5.65 ± 0.52	0.860

*Note:* a, b, and c represent that there exists statistically different with the gradually decreasing group, the steadily decreasing group, and the steadily increasing group, respectively.

**Table 3:** Results of mixed-effect model with gradually decreasing group as the reference.

Characteristics	Steadily increasing vs. gradually decreasing			Steadily decreasing vs. gradually decreasing			Gradually increasing vs. gradually decreasing		
	OR	95% CI	*p* values	OR	95% CI	*p* values	OR	OR	*p* values
BMI	1.42	(1.36, 1.48)	< 0.001	1.21	(1.16, 1.26)	< 0.001	1.67	(1.509, 1.836)	< 0.001
FBG	0.86	(0.79, 0.93)	< 0.001	0.85	(0.803, 0.904)	< 0.001	0.793	(0.666, 0.948)	< 0.001
TG	5.02	(4.36, 5.74)	< 0.001	3.03	(2.69, 3.41)	< 0.001	4.39	(3.536, 5.456)	< 0.001
HDL-C	0.001	(0.00043, 0.0011)	< 0.001	0.02	(0.013, 0.032)	< 0.001	0.001	(0.000005, 0.00013)	< 0.001
AST	1.01	(1.00216, 1.01797)	< 0.001	—	—	—	1.02	(1.0104, 1.0508)	< 0.001
Urine pH	—	—	—	1.19	(1.06, 1.34)	< 0.001	—	—	

**Table 4:** Results of mixed-effect model with steadily decreasing or steadily increasing group as the reference.

Characteristics	Steadily increasing vs. steadily decreasing			Gradually increasing vs. steadily decreasing			Gradually increasing vs. steadily increasing		
	OR	95% CI	*p* values	OR	95% CI	*p* values	OR	OR	*p* values
BMI	1.23	(1.210, 1.257)	< 0.001	1.419	(1.364, 1.476)	< 0.001	1.20	(1.15, 1.24)	< 0.001
FBG	1.07	(1.031, 1.115)	< 0.001	1.116	(1.032, 1.207)	< 0.001	1.07	(1.01, 1.14)	< 0.001
TG	2.096	(1.976, 2.221)	< 0.001	2.410	(2.185, 2.658)	< 0.001	1.35	(1.27, 1.43)	< 0.001
HDL-C	0.0093	(0.00043, 0.0011)	< 0.001	0.00027	(0.00012, 0.00055)	< 0.001	0.017	(0.010, 0.028)	< 0.001
BUN	1.01	(0.0070, 0.0124)	< 0.001	—	—	—	0.89	(0.82, 0.96)	< 0.001
Total serum cholesterol	—	—	—	0.870	(0.787, 0.959)	< 0.001	—	—	

**Table 5:** The comparison of prediction models.

Dataset	Model	Accuracy	Kappa	Sensitivity	Specificity	Precision	Recall	F1-score	AUC
Training set	SVM	0.801	0.601	0.823	0.778	0.804	0.823	0.813	0.879
	XGBoost	0.798	0.595	0.842	0.751	0.789	0.842	0.814	0.864
	Random forest	0.822	0.624	0.827	0.814	0.831	0.827	0.829	0.874
	KNN	0.575	0.144	0.641	0.502	0.588	0.641	0.613	0.731
	LR	0.793	0.586	0.813	0.772	0.798	0.813	0.805	0.822

Test set	SVM	0.786	0.571	0.778	0.794	0.798	0.794	0.761	0.814
	XGBoost	0.791	0.581	0.799	0.783	0.802	0.799	0.801	0.724
	Random forest	0.812	0.623	0.779	0.847	0.848	0.779	0.813	0.852
	KNN	0.577	0.149	0.645	0.502	0.589	0.645	0.616	0.786
	LR	0.773	0.546	0.789	0.756	0.781	0.789	0.785	0.878

## Data Availability

The dataset used in the present study can be obtained by contacting the authors Xiaona Cong and Yansu Chen under reasonable requires.

## Nomenclature

WHO: World Health Organization
MetS: Metabolic syndrome
LGMM: Latent growth mixed model
BMI: Body mass index
SBP: Systolic blood pressure
DBP: Diastolic blood pressure
FBG: Fasting blood glucose
TG: Triglyceride
HDL-C: High-density lipoprotein cholesterol
LDL-C: Low-density lipoprotein cholesterol
WBC: White blood cell count
RBC: Red blood cell count
ALT: Alanine aminotransferase
AST: Aspartate aminotransferase
GGT: *γ*-glutamyltransferase
BUN: Blood urea nitrogen
cMetS-S: Continuous metabolic syndrome severity score
CFA: Confirmatory factor analysis
T2DM: Type 2 diabetes mellitus
AIC: Akaike Information Criterion
BIC: Bayesian Information Criterion
APP: Average posterior probability
OR: Odds ratio
SVM: Support vector machine
AUC: Area under the curve
ROC: Receiver operating characteristic
SD: Standard deviation
ANOVA: Analysis of variance

## Supporting Information

Supporting Table S1. General description of the data from the physical examination of the study population.

Supporting Table S2. MetS developmental trajectory fitting.

Supporting Table S3. Lifestyle questionnaire data from railway employees.

Supporting Table S4. Model-fit indices for trajectory solutions based on lifestyle questionnaire variables.

Supporting Figure S1. Variable importance scores derived from Boruta feature selection after incorporating questionnaire variables.

Supporting Figure S2. SHAP feature importance plot generated from the optimal XGBoost model.

Supporting Figure S3. SHAP bee-swarm plot illustrating feature effects based on the optimal XGBoost model.

## References

[1] Xi J., Li P. W., Yu D. S. (2024). Multimorbidity: The Need for a Consensus on its Operational Definition. *Journal of Advanced Nursing*.

[2] Lu J., Wang Y., Hou L., Zuo Z., Zhang N., Wei A. (2021). Multimorbidity Patterns in Old Adults and Their Associated Multi-Layered Factors: A Cross-Sectional Study. *BMC Geriatrics*.

[3] Han S., Li S., Yang Y. (2024). Mapping Multimorbidity Progression Among 190 Diseases. *Communications Medicine*.

[4] Li S., Wen C. P., Tu H. (2025). Metabolic Syndrome Including Both Elevated Blood Pressure and Elevated Fasting Plasma Glucose is Associated With Higher Mortality Risk: A Prospective Study. *Diabetology & Metabolic Syndrome*.

[5] Tang X., Liu S., Qiu X. (2024). High Prevalence of Hyperuricemia and the Association With Metabolic Syndrome in the Rural Areas of Southwestern China: A Structural Equation Modeling Based on the Zhuang Minority Cohort. *Nutrition, Metabolism, and Cardiovascular Diseases*.

[6] Moradkhani A., Mohammadzadeh P., Assadi S., Saed L., Baradaran H. R., Moradi Y. (2025). Prevalence of Metabolic Syndrome and its Components in Iran: An Updated Meta-Analysis. *BMC Endocrine Disorders*.

[7] Xu H., Li X., Adams H., Kubena K., Guo S. (2018). Etiology of Metabolic Syndrome and Dietary Intervention. *International Journal of Molecular Sciences*.

[8] Lann D., Leroith D. (2007). Insulin Resistance as the Underlying Cause for the Metabolic Syndrome. *Medical Clinics of North America*.

[9] Rochlani Y., Pothineni N. V., Kovelamudi S., Mehta J. L. (2017). Metabolic Syndrome: Pathophysiology, Management, and Modulation by Natural Compounds. *Therapeutic Advances in Cardiovascular Disease*.

[10] Qin X., Qiu L., Tang G. (2020). Prevalence of Metabolic Syndrome Among Ethnic Groups in China. *BMC Public Health*.

[11] Canal-Rivero M., Ruiz-Veguilla M., Ortiz-García de la Foz V. (2023). Longitudinal Trajectories in Negative Symptoms and Changes in Brain Cortical Thickness: 10-Year Follow-Up Study. *The British Journal of Psychiatry: The Journal of Mental Science*.

[12] Li W., Jin C., Vaidya A. (2017). Blood Pressure Trajectories and the Risk of Intracerebral Hemorrhage and Cerebral Infarction: A Prospective Study. *Hypertension (DallasHypertension, Tex)*.

[13] Amouzegar A., Honarvar M., Masoumi S., Khalili D., Azizi F., Mehran L. (2023). Trajectory Patterns of Metabolic Syndrome Severity Score and Risk of Type 2 Diabetes. *Journal of Translational Medicine*.

[14] Honarvar M., Masoumi S., Mehran L., Khalili D., Amouzegar A., Azizi F. (2023). Development and Validation of a Continuous Metabolic Syndrome Severity Score in the Tehran Lipid and Glucose Study. *Scientific Reports*.

[15] Gurka M. J., Filipp S. L., Musani S. K., Sims M., Deboer M. D. (2018). Use of BMI as the Marker of Adiposity in a Metabolic Syndrome Severity Score: Derivation and Validation in Predicting Long-Term Disease Outcomes. *Metabolism*.

[16] Deng L., Liu T., Liu C. A. (2024). The Association of Metabolic Syndrome Score Trajectory Patterns With Risk of all Cancer Types. *Cancer*.

[17] Lawlor D. A., Ebrahim S., May M., Davey Smith G. (2004). (Mis) Use of Factor Analysis in the Study of Insulin Resistance Syndrome. *American Journal of Epidemiology*.

[18] Li C., Ford E. S. (2007). Is There a Single Underlying Factor for the Metabolic Syndrome in Adolescents? A Confirmatory Factor Analysis. *Diabetes Care*.

[19] Solomonov N., Lee J., Banerjee S. (2021). Modifiable Predictors of Nonresponse to Psychotherapies for Late-Life Depression With Executive Dysfunction: A Machine Learning Approach. *Molecular Psychiatry*.

[20] Mughal M. K., Giallo R., Arshad M. (2023). Trajectories of Maternal Depressive Symptoms From Pregnancy to 11 Years Postpartum: Findings From Avon Longitudinal Study of Parents and Children (ALSPAC) Cohort. *Journal of Affective Disorders*.

[21] Depaoli S., van de Schoot R., van Loey N., Sijbrandij M. (2015). Using Bayesian Statistics for Modeling Ptsd Through Latent Growth Mixture Modeling: Implementation and Discussion. *European Journal of Psychotraumatology*.

[22] Galatzer-Levy I. R. (2015). Applications of Latent Growth Mixture Modeling and Allied Methods to Posttraumatic Stress Response Data. *European Journal of Psychotraumatology*.

[23] Che B., Zhong C., Zhang R. (2023). Triglyceride-Glucose Index and Triglyceride to High-Density Lipoprotein Cholesterol Ratio as Potential Cardiovascular Disease Risk Factors: An Analysis of UK Biobank Data. *Cardiovascular Diabetology*.

[24] Berardi G., Cucchetti A., Sposito C. (2024). Recurrence and Tumor-Related Death After Resection of Hepatocellular Carcinoma in Patients With Metabolic Syndrome. *Jhep Reports*.

[25] Hassanloo N., Mehran L., Amouzegar A. (2024). Association Between Body Mass Index Trajectories and Type 2 Diabetes Incidence Over an 18-Year Follow-Up in the Tehran Lipid and Glucose Study. *Scientific Reports*.

[26] Ying M., Hu X., Li Q., Dong H., Zhou Y., Chen Z. (2022). Long-Term Trajectories of BMI and Cumulative Incident Metabolic Syndrome: A Cohort Study. *Frontiers in Endocrinology*.

[27] Guo T., Zheng S., Chen T. (2024). The Association of Long-Term Trajectories of BMI, Its Variability, and Metabolic Syndrome: A 30-Year Prospective Cohort Study. *EclinicalMedicine*.

[28] Frank D. M., Bradshaw P. T., Mujahid M., Epel E., Lararia B. A. (2023). Adolescent BMI Trajectory and Associations With Adult Metabolic Syndrome and Offspring Obesity. *Obesity*.

[29] Tarcău B. M., Vicaş L. G., Filip L. (2023). Emerging Perspectives on the Set of Conditions That Lead to the Emergence of Metabolic Syndrome. *Journal of Personalized Medicine*.

[30] Sookoian S., Pirola C. J. (2015). Liver Enzymes, Metabolomics and Genome-Wide Association Studies: From Systems Biology to the Personalized Medicine. *World Journal of Gastroenterology*.

[31] Shimodaira M., Okaniwa S., Nakayama T. (2017). Fasting Single-Spot Urine pH Is Associated With Metabolic Syndrome in the Japanese Population. *Medical Principles and Practice*.

[32] Yanai H., Adachi H., Hakoshima M., Katsuyama H. (2021). Molecular Biological and Clinical Understanding of the Pathophysiology and Treatments of Hyperuricemia and its Association With Metabolic Syndrome, Cardiovascular Diseases and Chronic Kidney Disease. *International Journal of Molecular Sciences*.

[33] Scurt F. G., Ganz M. J., Herzog C., Bose K., Mertens P. R., Chatzikyrkou C. (2024). Association of Metabolic Syndrome and Chronic Kidney Disease. *Obesity Reviews*.

