# Supplementary material for: Identifying Four Developmental Trajectories of Metabolic Syndrome and Their Influencing Factors: A Longitudinal Cohort Study of Railway Employees' Physical Examinations
Source: Int J Endocrinol. 2025 Nov 14;2025:9237368. doi: 10.1155/ije/9237368 (PMC12638151; doi:10.1155/ije/9237368)
Supplement: Supporting Information — Additional supporting information can be found online in the Supporting Information section. [file 9237368.f1.docx]

**Supporting Information**

**Identifying four developmental trajectories of metabolic syndrome and their influence factors: A longitudinal cohort of railway employees’ medical examinations**

**Table S1 General description of the data from the physical examination of the study population**

| **Characteristic** | **Value (mean± SD)** | **The normal range** |
| --- | --- | --- |
| Weight (kg) | 84.68±9.20 | / |
| Height (m) | 1.72 ± 0.06 | / |
| BMI (kg/m2) | 28.40 ± 2.25 | BMI<24 |
| SBP (mmHg) | 147.65 ± 14.09 | <140 |
| DBP (mmHg) | 94.90 ± 9.74 | <90 |
| Heart rate (/min) | 77.97 ± 9.63 | 60 - 100 |
| FBG (mmol/L) | 6.26 ± 1.14 | <6.1 |
| TG (mmol/L) | 2.92 ± 1.08 | <1.7 |
| Total cholesterol (μmol/L) | 5.21 ± 0.90 | <5.2 |
| HDL-C (mmol/L) | 1.20 ± 0.23 | ≥1.04 |
| Low-density lipoprotein (mmol/L) | 2.85 ± 0.78 | <3.4 |
| Haemoglobin (g/L) | 157.87 ± 9.91 | 120 - 160(male) |
| Red blood cells (10^12/L) | 5.12 ± 0.36 | 4.0 - 5.5×10^12(male) |
| White blood cells (10^9/L) | 6.97 ± 1.51 | 4.0 - 10.0×10^9 |
| Alanine aminotransferase (U/L) | 22.89 ± 6.58 | <40 |
| Aspartate aminotransferase (U/L) | 30.23 ± 13.23 | <40 |
| γ-glutamyltransferase (U/L) | 51.43 ± 25.82 | <50 |
| Total bilirubin (μmol/L) | 13.00 ± 4.38 | <21 |
| Direct bilirubin (μmol/L) | 3.48 ± 1.34 | <6.8 |
| Total protein (g/L) | 74.18 ± 4.31 | 60 - 80 |
| Albumin (g/L) | 45.93 ± 2.83 | 35 - 55 |
| Blood urea nitrogen (mmol/L) | 5.17 ± 1.13 | 2.9 - 7.8 |
| Uric acid (μmol/L) | 379.04 ± 63.46 | 149 - 416(male) |
| Creatinine (μmol/L) | 70.03 ± 0.17 | 53 - 106(male) |
| Urine pH | 5.63 ± 0.61 | 4.5 - 8.0 |

**Table S2 MetS developmental trajectory fitting**

| **Parameters** | **Number of trajectory fits** | | | | |
| --- | --- | --- | --- | --- | --- |
|  | 1 | 2 | 3 | 4 | 5 |
| **BIC** | 25946.01 | 25743.80 | 25726.10 | 25718.45 | 25755.91 |
| **AIC** | 25906.97 | 26576.86 | 25617.79 | 25595.74 | 25605.31 |
| **Log-likelihood** | -12946.48 | -12826.43 | -12791.89 | -12775.87 | -12775.66 |
| **APP** |  |  |  |  |  |
| Group 1 | 1.00 | 0.80 | 0.81 | 0.79 | 0.78 |
| Group 2 | - | 0.82 | 0.80 | 0.83 | 0.70 |
| Group 3 | - | - | 0.74 | 0.75 | 0.00 |
| Group 4 | - | - | - | 0.77 | 0.72 |
| Group 5 | - | - | - | - | 0.83 |
| **Proportion (%)** |  |  |  |  |  |
| Group 1 | 100.00 | 42.78 | 12.18 | 7.11 | 6.85 |
| Group 2 | - | 57.21 | 31.88 | 10.44 | 39.91 |
| Group 3 | - | - | 55.93 | 40.48 | 0.00 |
| Group 4 | - | - | - | 41.96 | 42.78 |
| Group 5 | - | - | - | - | 10.44 |

**Table S3 Lifestyle questionnaire data from railway employees**

| Variables | Total (n = 1073) | Gradually decreasing group (n = 79) | Steadily decreasing group (n = 106) | Steadily increasing group (n = 426) | Gradually increasing group (n = 462) | χ² | P |
| --- | --- | --- | --- | --- | --- | --- | --- |
|  |  |  |  |  |  |  |  |
| Work age |  |  |  |  |  | 7.69 | <.001 |
| ＜15years | 72 (6.71) | 17 (23.61) | 2 (2.78) | 34 (47.22) | 19 (26.39) |  |  |
| ≥15years | 1001 (93.29) | 62 (6.19) | 104 (10.39) | 392 (39.16) | 443 (44.26) |  |  |
| Smoking |  |  |  |  |  | 5.01 | 0.171 |
| no | 455 (42.40) | 27 (5.93) | 49 (10.77) | 171 (37.58) | 208 (45.71) |  |  |
| yes | 618 (57.60) | 52 (8.41) | 57 (9.22) | 255 (41.26) | 254 (41.10) |  |  |
| METs |  |  |  |  |  | 2.65 | 0.448 |
| good | 708 (65.98) | 46 (6.50) | 69 (9.75) | 288 (40.68) | 305 (43.08) |  |  |
| poor | 365 (34.02) | 33 (9.04) | 37 (10.14) | 138 (37.81) | 157 (43.01) |  |  |
| Drinking |  |  |  |  |  | 3.67 | 0.299 |
| no | 254 (23.67) | 23 (9.06) | 23 (9.06) | 109 (42.91) | 99 (38.98) |  |  |
| yes | 819 (76.33) | 56 (6.84) | 83 (10.13) | 317 (38.71) | 363 (44.32) |  |  |
| Marital status |  |  |  |  |  | 3.76 | 0.289 |
| no | 986 (91.89) | 69 (7.00) | 99 (10.04) | 388 (39.35) | 430 (43.61) |  |  |
| yes | 87 (8.11) | 10 (11.49) | 7 (8.05) | 38 (43.68) | 32 (36.78) |  |  |
| Work location |  |  |  |  |  | 1.94 | 0.584 |
| inside | 355 (33.08) | 31 (8.73) | 32 (9.01) | 143 (40.28) | 149 (41.97) |  |  |
| outside | 718 (66.92) | 48 (6.69) | 74 (10.31) | 283 (39.42) | 313 (43.59) |  |  |
| Work shift |  |  |  |  |  | 2.30 | 0.513 |
| day shift | 388 (36.16) | 24 (6.19) | 34 (8.76) | 158 (40.72) | 172 (44.33) |  |  |
| night shift | 685 (63.84) | 55 (8.03) | 72 (10.51) | 268 (39.12) | 290 (42.34) |  |  |
| Living arrangement |  |  |  |  |  | 9.08 | 0.028 |
| with someone | 876 (81.64) | 55 (6.28) | 91 (10.39) | 351 (40.07) | 379 (43.26) |  |  |
| alone | 197 (18.36) | 24 (12.18) | 15 (7.61) | 75 (38.07) | 83 (42.13) |  |  |
| Drinking tea |  |  |  |  |  | χ²=2.77 | 0.429 |
| no | 119 (11.09) | 8 (6.72) | 15 (12.61) | 52 (43.70) | 44 (36.97) |  |  |
| yes | 954 (88.91) | 71 (7.44) | 91 (9.54) | 374 (39.20) | 418 (43.82) |  |  |
| Daily water intake |  |  |  |  |  | 1.89 | 0.596 |
| ＜500ml | 354 (32.99) | 21 (5.93) | 33 (9.32) | 144 (40.68) | 156 (44.07) |  |  |
| ≥500ml | 719 (67.01) | 58 (8.07) | 73 (10.15) | 282 (39.22) | 306 (42.56) |  |  |
| Consume spicy food |  |  |  |  |  | 4.27 | 0.234 |
| no | 411 (38.30) | 32 (7.79) | 40 (9.73) | 148 (36.01) | 191 (46.47) |  |  |
| yes | 662 (61.70) | 47 (7.10) | 66 (9.97) | 278 (41.99) | 271 (40.94) |  |  |
| Daily salt intake |  |  |  |  |  | 3.43 | 0.331 |
| ＜5g | 241 (22.46) | 12 (4.98) | 21 (8.71) | 102 (42.32) | 106 (43.98) |  |  |
| ≥5g | 832 (77.54) | 67 (8.05) | 85 (10.22) | 324 (38.94) | 356 (42.79) |  |  |
| Daily oil intake |  |  |  |  |  | 3.06 | 0.382 |
| ＜8g | 1020 (95.06) | 72 (7.06) | 101 (9.90) | 408 (40.00) | 439 (43.04) |  |  |
| ≥8g | 53 (4.94) | 7 (13.21) | 5 (9.43) | 18 (33.96) | 23 (43.40) |  |  |
| Late-night eating |  |  |  |  |  | 28.04 | <.001 |
| no | 585 (54.52) | 23 (3.93) | 68 (11.62) | 224 (38.29) | 270 (46.15) |  |  |
| yes | 488 (45.48) | 56 (11.48) | 38 (7.79) | 202 (41.39) | 192 (39.34) |  |  |
| Family history of hypertension |  |  |  |  |  | 2.32 | 0.509 |
| no | 611 (56.94) | 41 (6.71) | 66 (10.80) | 238 (38.95) | 266 (43.54) |  |  |
| yes | 462 (43.06) | 38 (8.23) | 40 (8.66) | 188 (40.69) | 196 (42.42) |  |  |

**#**METs were categorized as "good" when the percentile was >66. Post-hoc observed frequencies are presented as column percentages of the corresponding headcount.

**Table S4** Model-fit indices for trajectory solutions based on lifestyle questionnaire variables

| Metric | SVM | XGB | RF | DT | LR | ANN | NB | KNN |
| --- | --- | --- | --- | --- | --- | --- | --- | --- |
| Mean Accuracy | 0.7461 | 0.7121 | 0.7664 | 0.7109 | 0.6640 | 0.5962 | 0.5962 | 0.5962 |
| Mean AUC | 0.7695 | 0.7882 | 0.7973 | 0.6891 | 0.7594 | 0.6812 | 0.7853 | 0.6814 |
| Mean Precision | 0.7821 | 0.7912 | 0.7980 | 0.7651 | 0.7481 | 0.7771 | 0.7771 | 0.7771 |
| Mean Recall | 0.7522 | 0.7336 | 0.7192 | 0.7194 | 0.7013 | 0.7961 | 0.7961 | 0.7961 |
| Mean F1 | 0.6398 | 0.7146 | 0.7963 | 0.6478 | 0.6921 | 0.6274 | 0.6274 | 0.6274 |

**
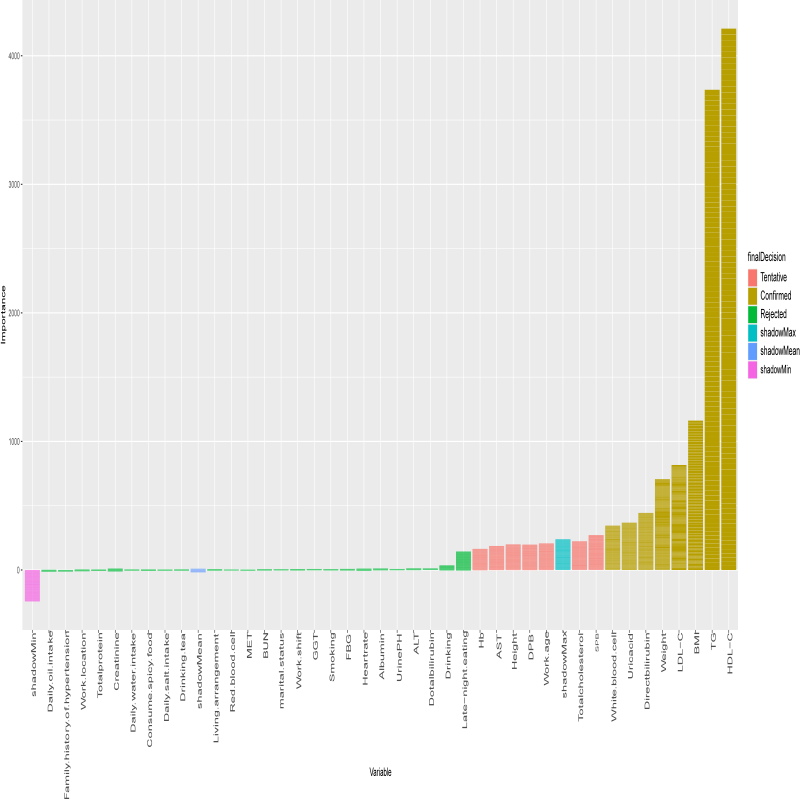
**

**Figure S1** Variable importance scores derived from Brouta feature selection after incorporating questionnaire variables.

**
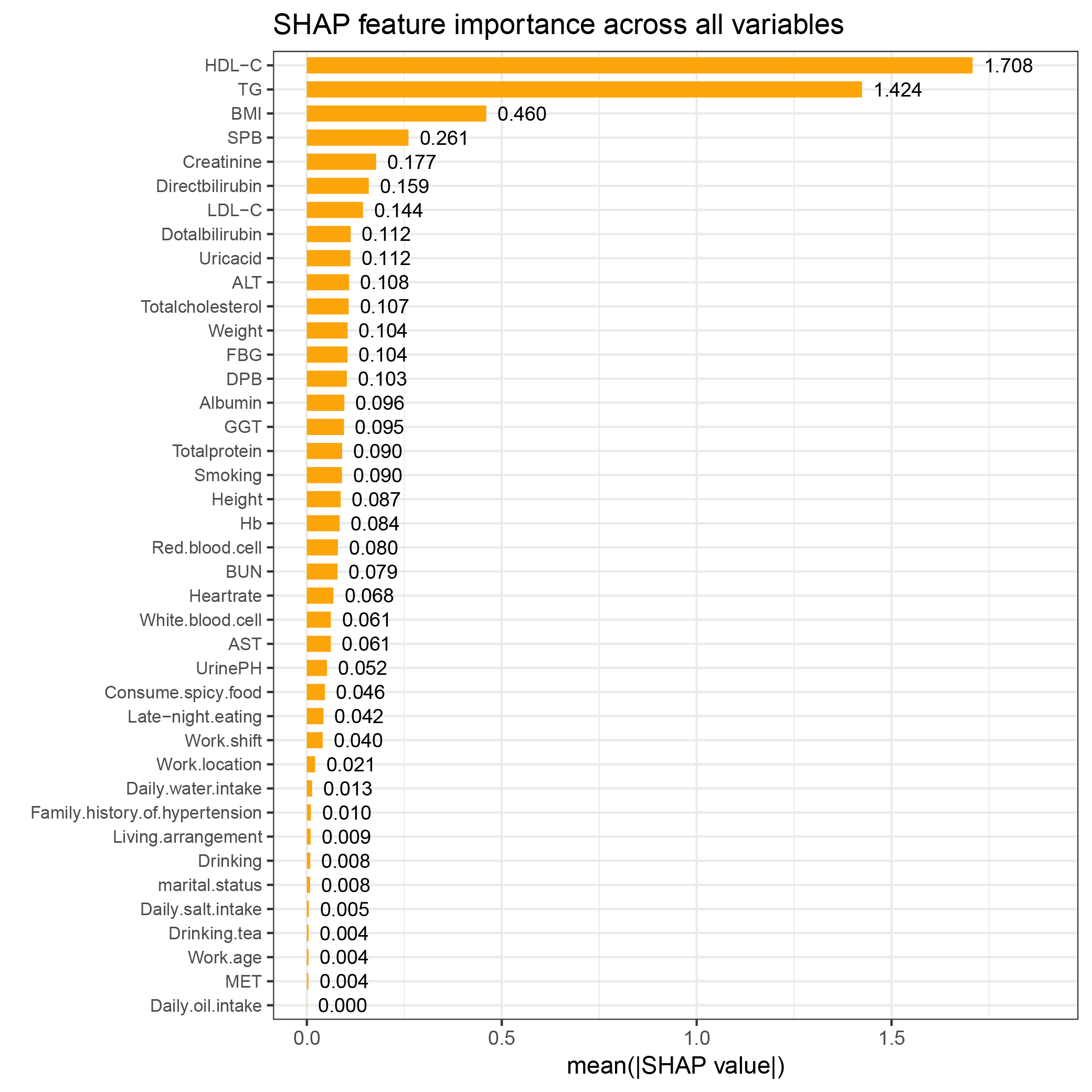
**

**Figure S2** SHAP feature importance plot generated from the optimal XGBoost model.

**
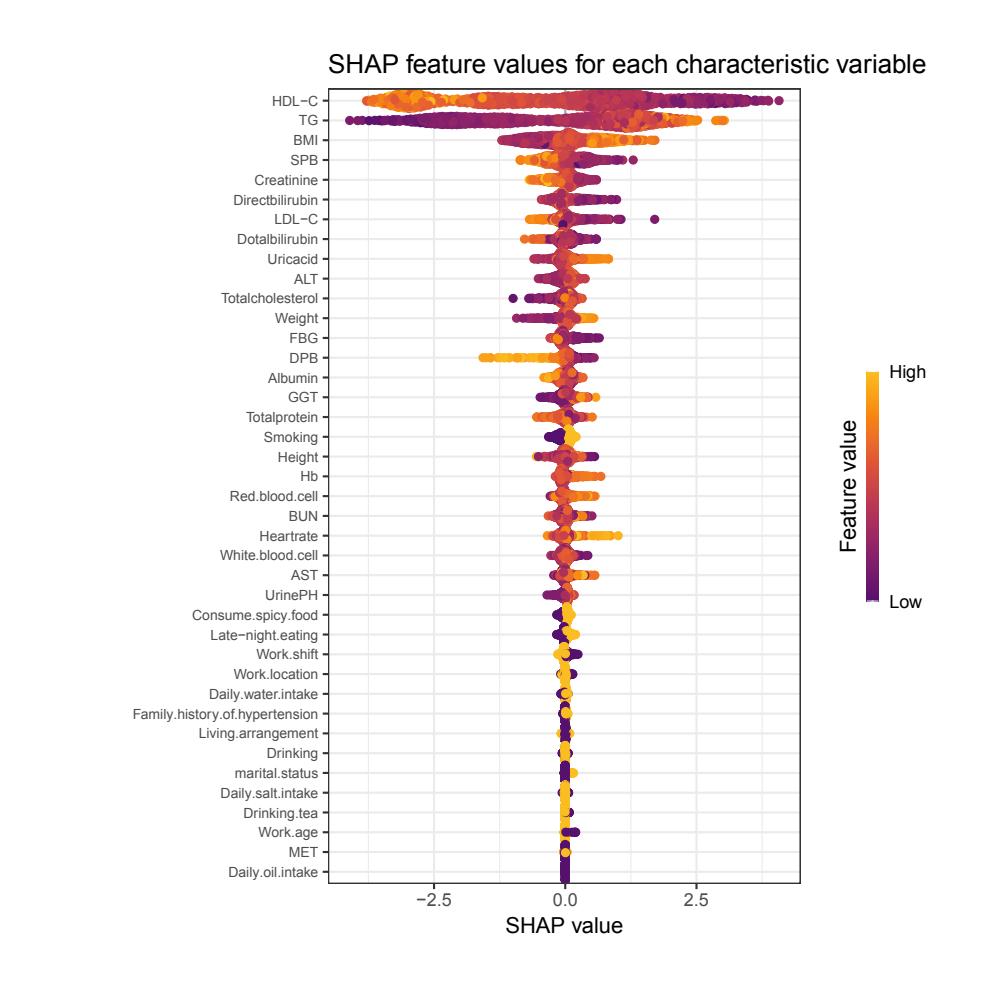
**

**Figure S3** SHAP beeswarm plot illustrating feature effects based on the optimal XGBoost model.
